# *Nlrp2*, a Maternal Effect Gene Required for Early Embryonic Development in the Mouse

**DOI:** 10.1371/journal.pone.0030344

**Published:** 2012-01-25

**Authors:** Hui Peng, Bohao Chang, Chenglong Lu, Jianmin Su, Yongyan Wu, Pin Lv, Yongsheng Wang, Jun Liu, Bowei Zhang, Fusheng Quan, Zekun Guo, Yong Zhang

**Affiliations:** 1 Key Laboratory of Animal Reproductive Physiology and Embryo Technology, College of Veterinary Medicine, Northwest Agriculture and Forestry University, Yangling, Shanxi, People's Republic of China; 2 Department of Biochemistry and Molecular Biology, College of Life Sciences, Northwest Agriculture and Forestry University, Yangling, Shanxi, People's Republic of China; McGill University, Canada

## Abstract

Maternal effect genes encode proteins that are produced during oogenesis and play an essential role during early embryogenesis. Genetic ablation of such genes in oocytes can result in female subfertility or infertility. Here we report a newly identified maternal effect gene, *Nlrp2*, which plays a role in early embryogenesis in the mouse. *Nlrp2* mRNAs and their proteins (∼118 KDa) are expressed in oocytes and granulosa cells during folliculogenesis. The transcripts show a striking decline in early preimplantation embryos before zygotic genome activation, but the proteins remain present through to the blastocyst stage. Immunogold electron microscopy revealed that the NLRP2 protein is located in the cytoplasm, nucleus and close to nuclear pores in the oocytes, as well as in the surrounding granulosa cells. Using RNA interference, we knocked down *Nlrp2* transcription specifically in mouse germinal vesicle oocytes. The knockdown oocytes could progress through the metaphase of meiosis I and emit the first polar body. However, the development of parthenogenetic embryos derived from Nlrp2 knockdown oocytes mainly blocked at the 2-cell stage. The maternal depletion of *Nlrp2* in zygotes led to early embryonic arrest. In addition, overexpression of *Nlrp2* in zygotes appears to lead to normal development, but increases blastomere apoptosis in blastocysts. These results provide the first evidence that *Nlrp2* is a member of the mammalian maternal effect genes and required for early embryonic development in the mouse.

## Introduction

Maternal factors, which are encoded by maternal effect genes and accumulate during oogenesis, are critical to support the development of the preimplantation embryo [1]. Maternal effect genes were first described in mammals in 2000 [2], [3], although some early pioneering investigations had been reported in *Drosophila* [4], [5], *Caenorhabditis elegans* [6] and *Xenopus* [7]. Several maternal effect genes that function in oogenesis [8], meiotic maturation [9], preimplantation [8], [10], [11], [12], [13], [14], [15], [16] and postimplantation development [17] have been identified in the mouse. However, knowledge of the maternal effect genes involved in female reproduction and early embryo development is still limited in mammals.

NLRP (Nucleotide-binding oligomerization domain, Leucine rich Repeat and Pyrin domain containing Proteins) is a subfamily of the newly described CATERPILLER (CAspase-recruitment domain (CARD) Transcription Enhancer, R (purine)-binding, Pyrin, Lots of LEucine Repeats) family of proteins with a nucleotide-binding domain and a leucine-rich region [18], [19]. The *Nlrp* gene family contains 20 members in the mouse. Recent studies have demonstrated that these genes play key roles in reproduction. For instance, the mouse *Nlrp5* gene, known to encode for the Maternal Antigen That Embryos Require (MATER) [20], was one of the earliest maternal effect genes characterized at the molecular level in the mouse. Although showing fairly normal folliculogenesis, oocyte maturation and fertilization, *Nlrp5* knockout female mice are infertile because of a block of embryogenesis at the 2-cell stage [3]. Recently a subcortical maternal complex (SCMC), constituting MATER, FLOPED, TLE6 and FILIA has been identified. This assembles during oocyte growth, [15], [21]. It is essential for zygotes to progress beyond the first embryonic cell divisions. In vitro knockdown of *Nlrp14* in mouse zygotes leads to developmental arrest between the 2-cell and the 8-cell stages in more than 50% of the embryos [22]. Besides *Nlrp5* and *Nlrp14*, several other *Nlrp* genes also display specific or preferential oocyte expression patterns, the expression of which declines with oocyte aging [22], [23]. Additionally, in studies on the oocyte-to-embryo transition in mouse, *Nlrp2* (also known as *Nalp2/Pan1/Pypaf2*) was shown to be highly enriched in fully grown oocytes by reverse transcription polymerase chain reaction (RT–PCR) amplification [24]. Based on these limited previous studies, we hypothesized that *Nlrp2* would be essential for early embryo development and that perturbing its function in the oocyte and zygote would disrupt early embryogenesis.

To test this hypothesis, we employed an RNA interference (RNAi) approach to inhibit *Nlrp2* function specifically in mouse germinal vesicle (GV) oocytes and zygotes. This is an efficient method for inhibiting the function of maternally and zygotically expressed genes in oocytes and zygotes [25], [26], [27], [28]. Electroporation was used to deliver silencing RNA because it is one of the most effective ways of delivering double stranded (ds)RNA and small interfering (si)RNA into mouse oocytes and early embryos [29], [30]. In addition, we constructed an expression vector for *Nlrp2* and microinjected it into mouse zygotes.

We report here that the maternal depletion of Nlrp2 in GV-stage oocytes and zygotes results in early developmental arrest. However, overexpression of *Nlrp2* in zygotes appears to lead to normal development, but increases apoptosis in blastocyst. In addition, *Nlrp2* is not required for oocyte maturation. Thus, *Nlrp2* can be added to the increasing—if limited—list of mammalian maternal effect genes.

## Results

### *Nlrp2* mRNA Expression Levels

**Figure 1:**
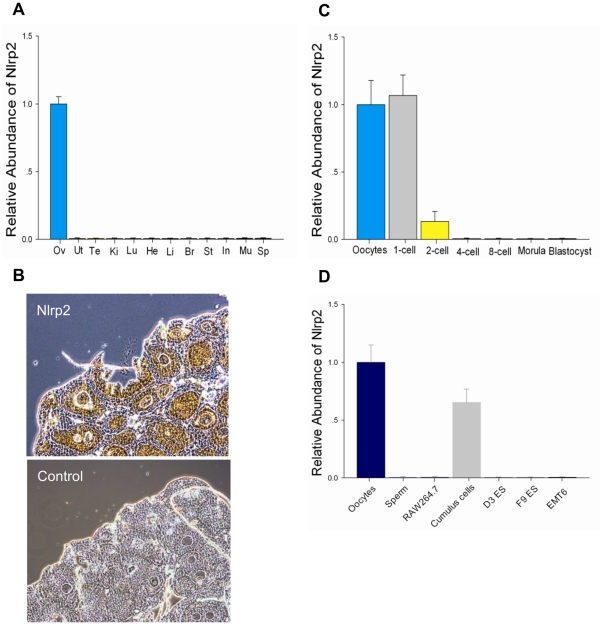
Developmental expression of *Nllrp2* in the mouse. (A) Quantitative reverse-transcription polymerase chain reaction (qRT–PCR) with total RNA extracted from 4-week-old mouse ovary (Ov), uterus (Ut), testis (Te), kidney (Ki), lung (Lu), heart (He), liver (Li), brain (Br), stomach (St), intestines (In), muscle (Mu), spleen (Sp) were performed. Results were normalized to the abundance in the ovary and expressed as the mean ± SEM. (B) In situ hybridization of fixed, paraffin wax-embedded 6 µm ovary sections probed with DIG-labeled *Nlrp2* oligonucleotide probes. The original magnification was ×100. (C) The relative abundance of *Nlrp2* transcripts in mouse oocytes and preimplantation embryos. (D) The relative abundance of *Nlrp2* transcripts in different mouse cells.

*Nlrp2* expression was detected in mouse ovaries, but not in eleven other tissues, including male testes (Fig. 1A). Within the ovary, expression was restricted to ovarian follicles at various stages (Fig. 1B). After ovulation and fertilization, the transcripts were immediately downregulated and not detected after the 2-cell stage during preimplantation development (Fig. 1C). In addition, except for oocytes and cumulus cells, *Nlrp2* mRNA was not detected in other cell lines (Fig. 1D), although human *NLRP2* is expressed in various tumor lines [31].

### NLRP2 Protein Levels

**Figure 2:**
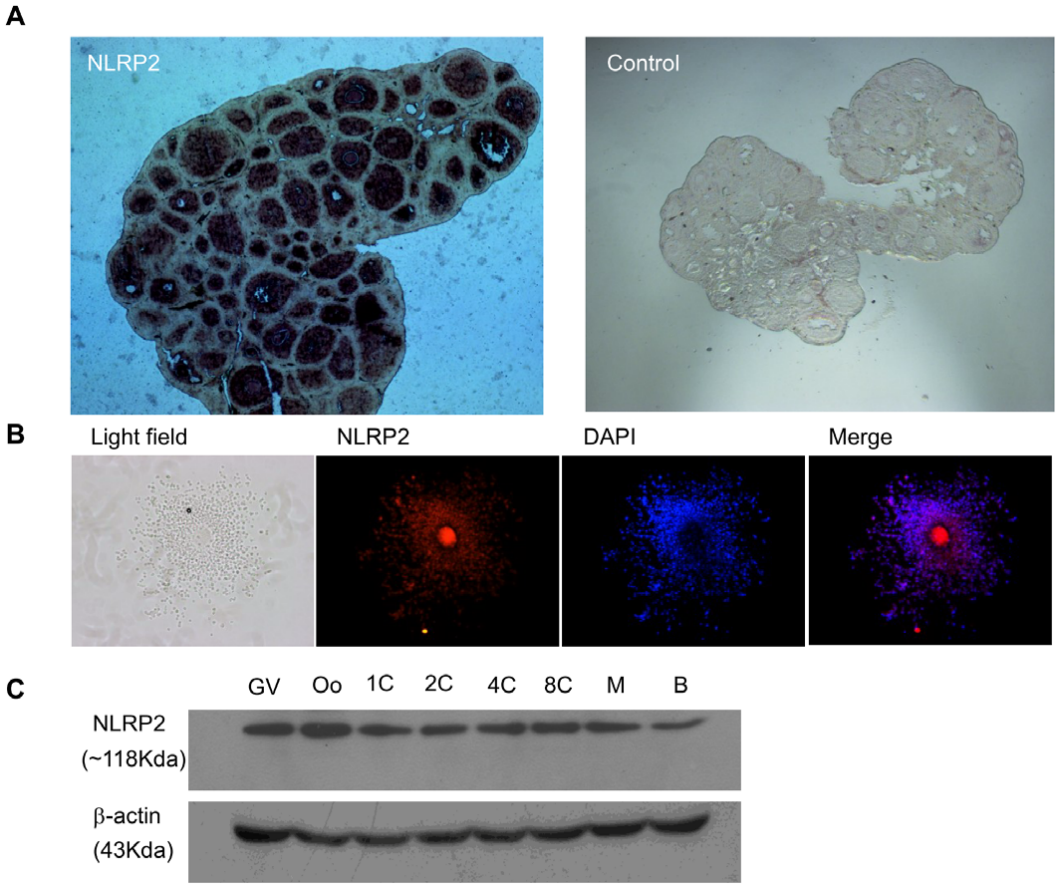
Developmental expression of NLRP2 protein in mouse. (A) Immunohistochemical analysis of sequential sections from a 3-week-old mouse ovary using an anti-NLRP2 antibody. The original magnification was ×40. (B) Immunofluorescent detection of NLRP2 in cumulus–oocyte complexes after permeabilization and incubation with an anti-NLRP2 antibody. The original magnification was ×100. (C) Immunoblots of lysates isolated from oocytes and preimplantation embryos. Molecular masses (KDa) are indicated on the left; β-actin was used as a control.

Immunohistochemistry, immunofluorescence and immunoblotting were used to assess expression at the protein level. Immunohistochemistry of ovarian sections suggested that the NLRP2 protein was expressed in oocytes and granulosa cells at various follicular stages (Fig. 2A). After ovulation and fertilization, the protein (∼118 KDa) was still present in cumulus–oocyte complexes (Fig. 2B) and remained present through the blastocyst stage (Fig. 2C) although *Nlrp2* mRNA was not detected after the 2-cell stage (Fig. 1C).

### Cellular and Subcellular Localization of NLRP2

**Figure 3:**
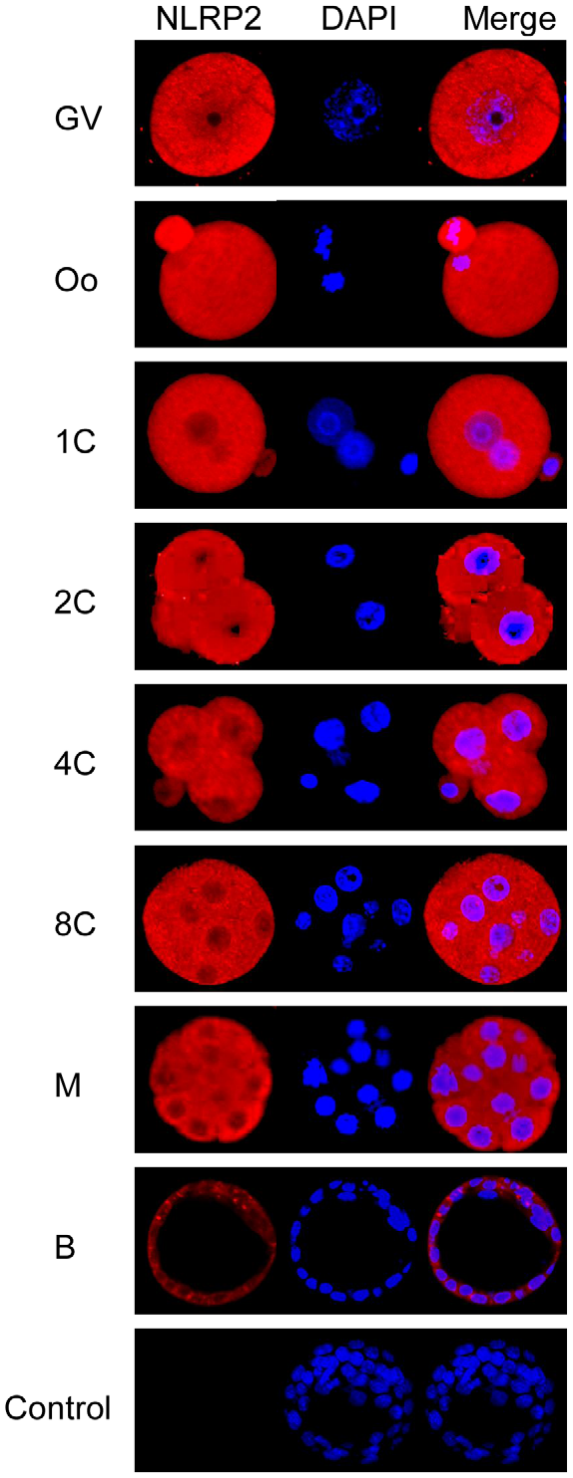
Cellular localization of NLRP2 protein. Confocal microscopic images of oocytes and preimplantation embryos. Each sample was counterstained with DAPI to visualize DNA (blue). The original magnification was ×200.

**Figure 4:**
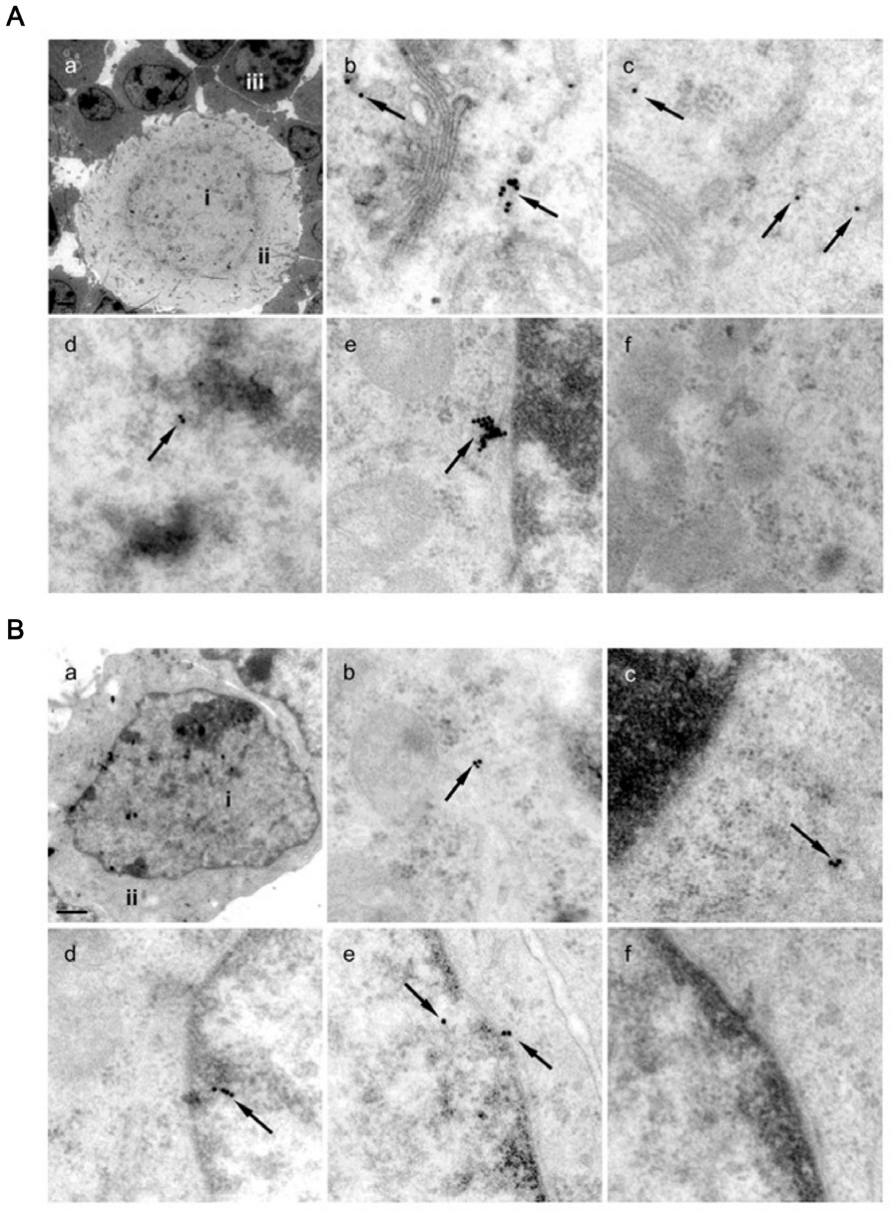
Subcellular localization of NLRP2 protein. (A) Subcellular localization of NLRP2 protein in immature mouse oocytes. Using an anti-NLRP2 antibody and ultrathin ovarian sections of 10-day-old mice, immunogold reactions were examined by transmission electron microscopy. Black spots with arrows are immunogold particles indicating the presence of NLRP2 protein. a, Oocytes and surrounding granulosa cells (×4,000). The positions of nucleus (i), cytoplasm (ii) and granulosa cells (iii) are indicated. b and c, Oocyte cytoplasm with immunogold particles (×50,000). d, Nucleus with immunogold particles (×50,000). e, Nuclear pore with immunogold particles nearby (×50,000). f, Control oocyte without immunogold particles in the absence of the primary antibody (×50,000). (B) Subcellular localization of NLRP2 protein in mouse granulosa cells. a, Granulosa cell (×15,000). The positions of the nucleus (i) and cytoplasm (ii). b and c, Cytoplasm with immunogold particles (×50,000). d, Nucleus with immunogold particles (×50,000). e, Nucleus and nuclear pore with immunogold particles (×50,000). f, Granulosa cell without immunogold particles in the absence of primary antibody (×50,000).

To determine the location of NLRP2 protein in oocytes and early embryos, we conducted confocal and immunogold electron microscopy analyses. Confocal microscopy demonstrated a predominant cytoplasmic location of the NLRP2 protein in oocytes and early embryos (Fig. 3). The distribution of NLRP2 protein in the subcellular organelles was monitored by immunogold staining and transmission electron microscopy. Immunogold particles were seen within the oocytes and the surrounding granulosa cells. They were mainly located in the cytoplasm, but some particles were present in the nucleus and close to nuclear pores in the oocytes (Fig. 4A), as well as the surrounding granulosa cells (Fig. 4B).

### Expression and Localization of *Nlrp2* in Parthenogenetic Embryos

**Figure 5:**
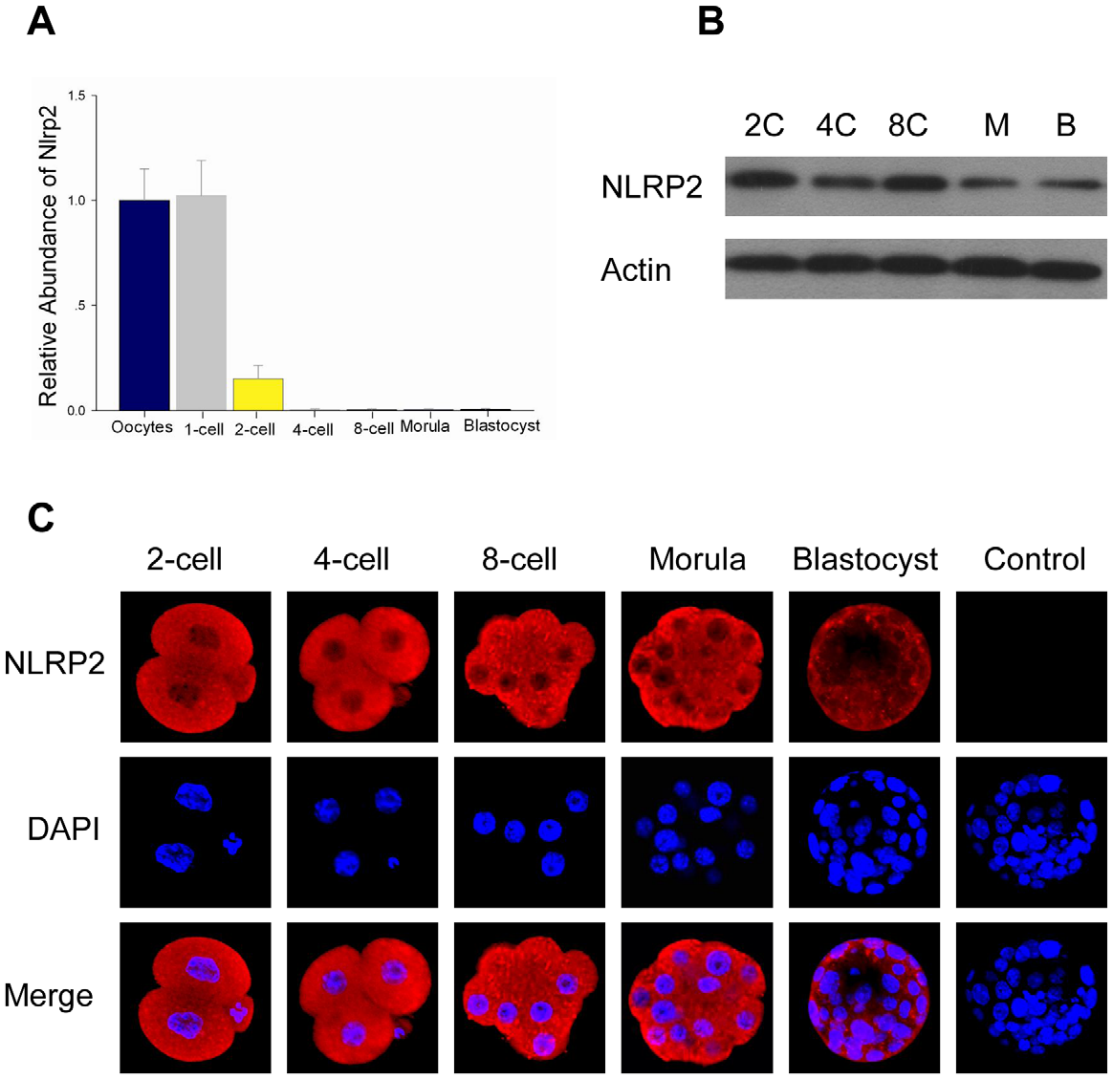
Developmental expression and localization of *Nlrp2* in parthenogenetic embryos. (A) The relative abundance of *Nlrp2* transcripts in mouse oocytes and parthenogenetic embryos. (B) Immunoblots of lysates isolated from parthenogenetic embryos. (C) Confocal microscopic images of parthenogenetic embryos. Each sample was counterstained with DAPI to visualize DNA (blue). The original magnification was ×200.

The amount of *Nlrp2* mRNA declined sharply after parthenogenetic activation of metaphase II oocytes (Fig. 5A). As the parthenogenetic embryos developed beyond the 2-cell stage, the transcripts virtually disappeared (Fig. 5A). However, the NLRP2 protein remained present at preimplantation stages (Fig. 5B). The protein was predominately localized in the blastomere cytoplasm (Fig. 5C). Thus, the expression profile of NLRP2 protein during embryogenesis, as determined by immunoblotting assay, was similar to that determined by confocal microscopic analysis.

### *Nlrp2* Is Not Required for Oocyte Maturation

**Figure 6:**
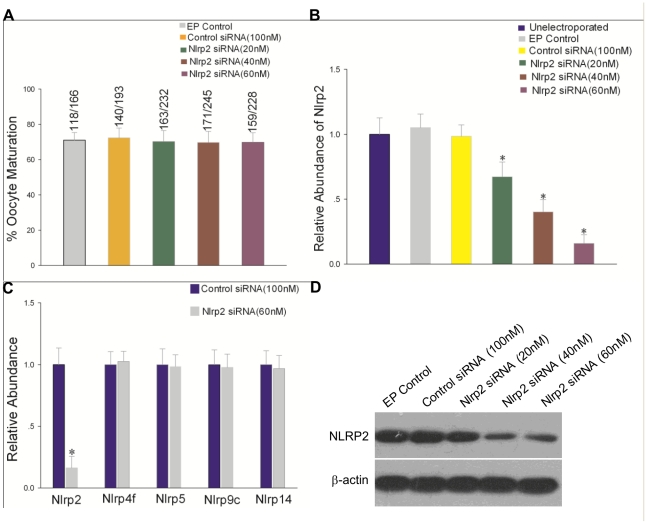
GV-stage oocyte maturation after electroporation with *Nlrp2* siRNA. (A) Oocyte maturation rate following electroporation (EP) of GV-stage oocytes in the presence or absence of control and *Nlrp2* siRNA. The numbers on top of each bar indicate the numbers of oocyte matured/numbers of oocytes electroporated. (B) The relative abundance of *Nlrp2* transcripts after electroporation with *Nlrp2* siRNA. The data have been normalized to untreated oocytes. Statistical comparisons were made using ANOVA and LSD tests (* p<0.05). (C) *Nlrp2*, *Nlrp4f*, *Nlrp5*, *Nlrp9c* and *Nlrp14* gene expression by qRT–PCR in mouse oocytes at 24 h after electroporation with *Nlrp2* siRNA (60 nM). Results were normalized to control siRNA (100 nM) group. * p<0.05. (D) Immunoblots of mouse oocytes at 24 h after electroporation in the presence or absence of control and *Nlrp2* siRNA.

Zona-weakened germinal vesicle (GV)-stage oocytes were electroporated with custom-made Nlrp2 siRNA mixture. As shown in Figure 6A, *Nlrp2* siRNA had no obvious effect on oocyte maturation. However, the *Nlrp2* gene was downregulated in a dose-dependent manner (Fig. 6B). The *Nlrp2* knockdown effects were specific as the expressions of *Nlrp4f*, *Nlrp5*, *Nlrp9c* and *Nlrp14*, randomly selected members of the *Nlrp* gene family associated with reproduction and embryo development, were not altered by the introduction of *Nlrp2* siRNA (60 nM) (Fig. 6C). Moreover, analysis of NLRP2 protein levels by immunoblotting showed that this obviously decreased in oocytes 24 h after electroporation with *Nlrp2* siRNA compared with control groups (Fig. 6D). Thus, the decline of *Nlrp2* mRNA and protein in oocytes after electroporation with *Nlrp2* siRNA did not affect oocyte maturation.

### *Nlrp2* Knockdown in Oocytes Leads to Early Embryonic Arrest After Parthenogenetic Activation

**Figure 7:**
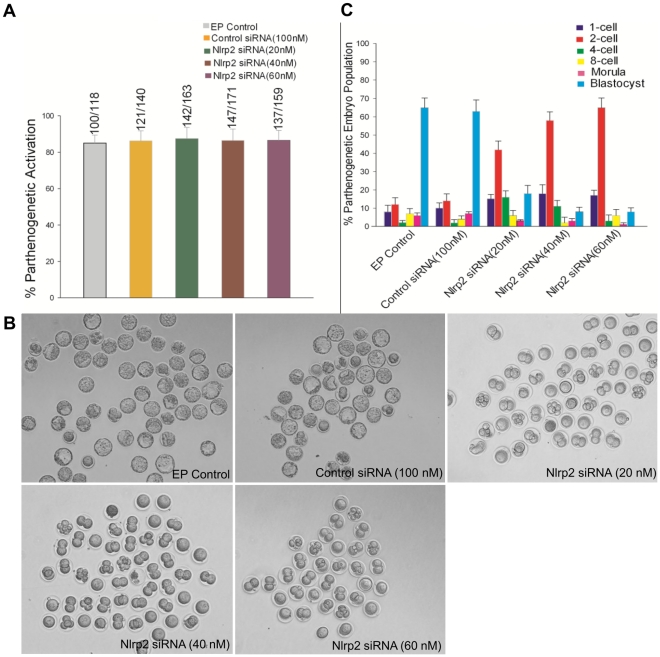
Development of parthenogenetic embryos derived from EP control, control siRNA and *Nlrp2* siRNA-treated groups (20, 40 and 60 nM). (A) Parthenogenetic activation rate of electroporated oocytes. (B) Morphological appearance of parthenogenetic embryos after being cultured for 3.5 days. The original magnification was ×100. (C) Percentage of parthenogenetic embryos at different stages after being cultured for 3.5 days.

Metaphase II oocytes (after extrusion of the first polar body) derived from oocytes electroporated at the GV stage were subjected to parthenogenetic activation and then cultured in KSOMaa-BSA medium (see Materials and Methods). There was no significant difference in the parthenogenetic activation rate of the oocytes obtained from the EP control, control siRNA or *Nlrp2* siRNA (20, 40 and 60 nM) groups (Fig. 7A). The embryonic developmental stages and their morphological appearance after being cultured for 3.5 days are presented in Figure 7B. The parthenogenetic embryos derived from the electroporation (EP) control and control siRNA reached the blastocyst stage at rates of 65% and 63%, respectively (Fig. 7C). However, the development of parthenogenetic embryos derived from *Nlrp2* siRNA-electroporated oocytes appeared to be blocked mainly at the 2-cell stage (Fig. 7C).

### *Nlrp2* Knockdown in Zygotes Causes Early Embryonic Arrest

**Figure 8:**
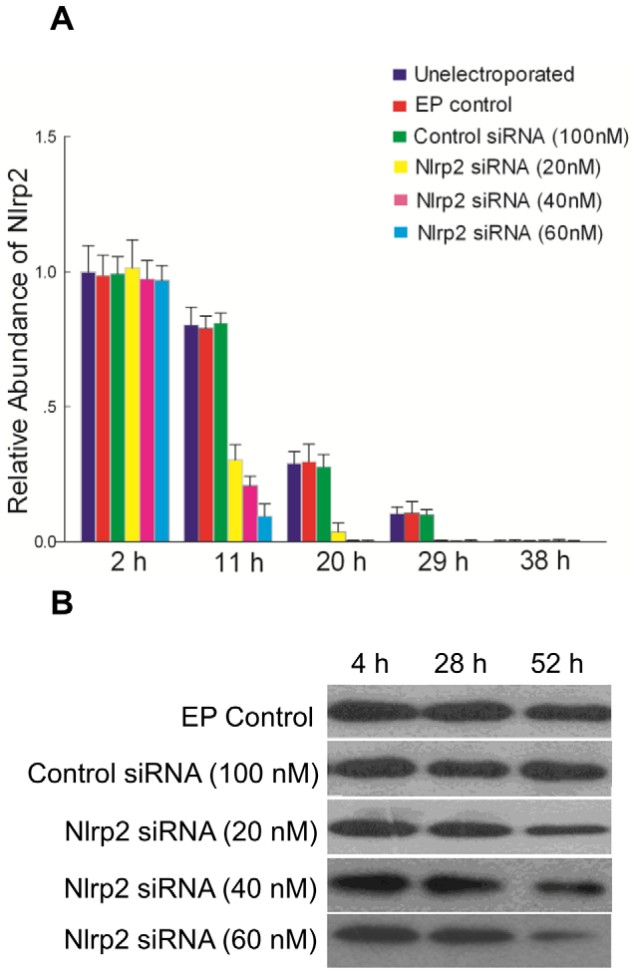
*Nlrp2* mRNA and protein levels in *Nlrp2* knockdown embryos. (A) The relative abundance of *Nlrp2* transcripts in mouse embryos collected at 2 h, 11 h, 20 h, 29 h and 38 h after electroporation. Results have been normalized to the abundance in untreated zygotes and are expressed as the mean ± SEM. (B) Immunoblots of mouse embryos at 4 h (1-cell), 28 h (2-cell) and 52 h (8-cell) after electroporation.

**Figure 9:**
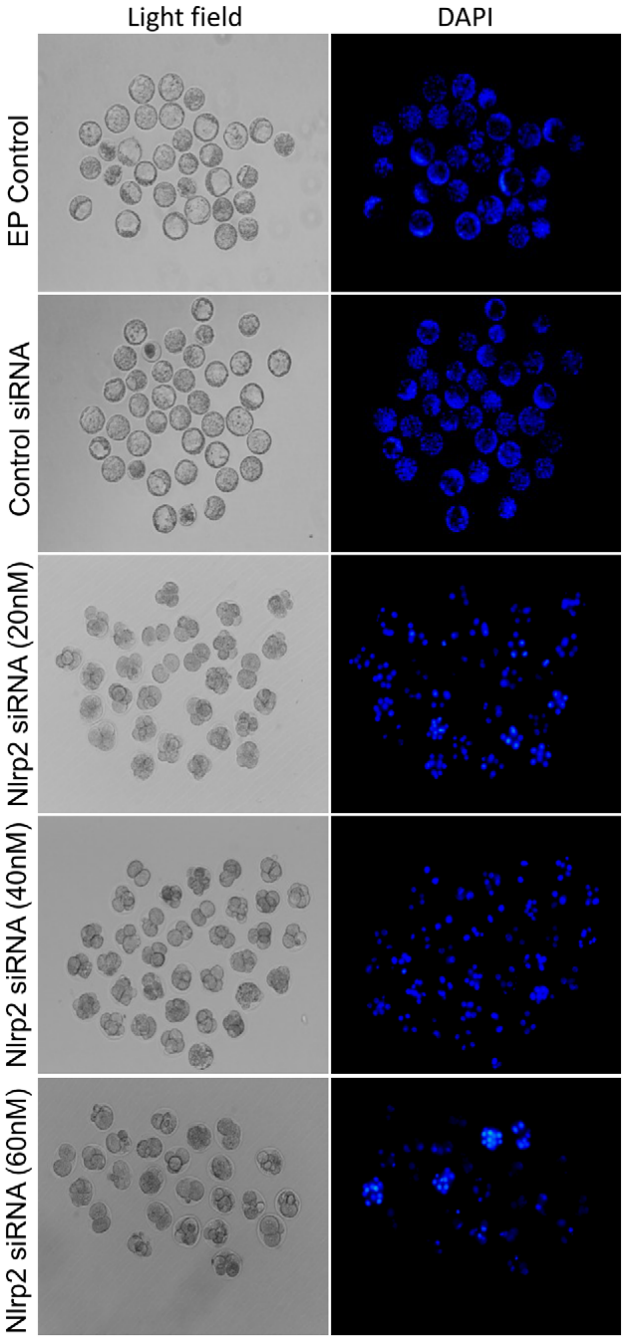
Morphology of *Nlrp2* knockdown embryos after being cultured for 3.5 days. Morphology (left) and DAPI staining (right). The original magnification was ×100.

**Figure 10:**
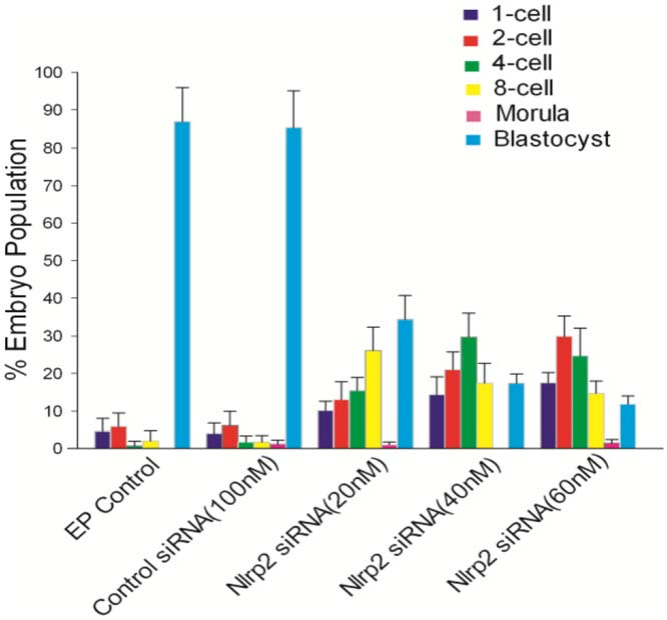
Developmental stages of *Nlrp2* knockdown embryos. Percentage of embryos at different stages after being cultured for 3.5 days.

To further confirm the function of *Nlrp2* in early embryonic development, the zygotes were electroporated with *Nlrp2* siRNA. As shown in Figure 8A, the introduction of *Nlrp2* siRNA resulted in the reduction of the targeted mRNA in time- and concentration-dependent manners. Moreover, it also led to a decrease in the NLRP2 protein level compared with control groups (Fig. 8B). Morphological analysis suggested that embryos derived from *Nlrp2* siRNA-electroporated zygotes appeared to be arrested, while the embryos derived from control groups reached the blastocyst stage (Fig. 9). The *Nlrp2* knockdown embryos were mainly arrested between the 2- and 8-cell stages (Fig. 10).

### Overexpression of *Nlrp2* in Zygotes Leads to Normal Development but Increased Apoptosis

**Figure 11:**
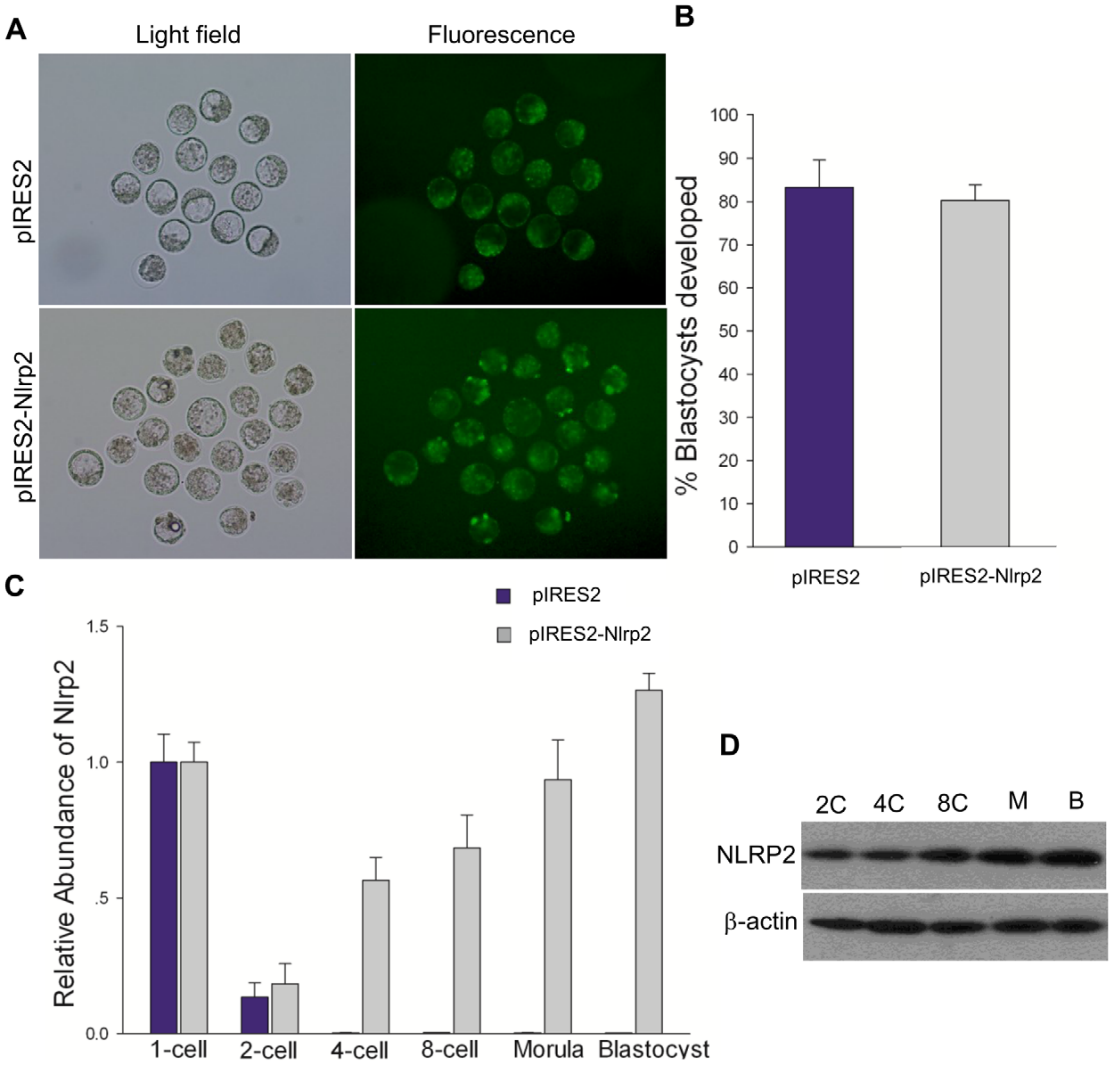
Overexpression of *Nlrp2* in zygotes. (A) Representative micrographs of blastocysts developing from zygotes that had been microinjected with pIRES2 or pIRES2-Nlrp2. Fluorescence images show the expression of GFP. The original magnification was ×100. (B) The blastocyst formation rate of zygotes microinjected with pIRES2 or pIRES2-Nlrp2. (C) The relative abundance of *Nlrp2* transcripts after microinjection. (D) Immunoblots of mouse embryos at different stages after microinjection.

**Figure 12:**
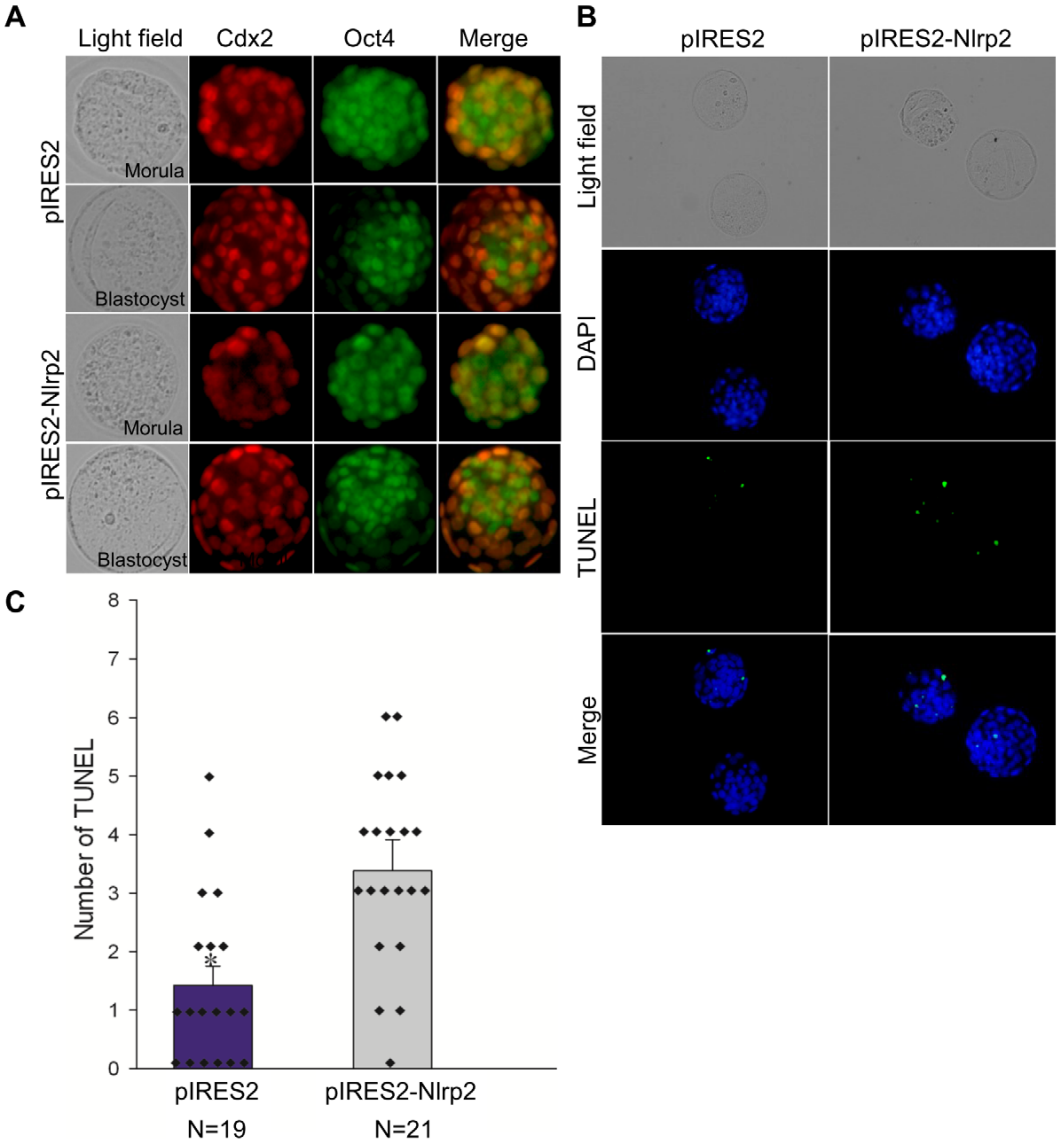
Effects of *Nlrp2* overexpression on the expression of CDX2 and OCT3/4 and incidence of apoptosis in blastocysts. (A) Immunostaining of CDX2 (red) and OCT3/4 (green) in morulae and blastocysts developing from zygotes that had been microinjected with pIRES2 or pIRES2-Nlrp2. The original magnification was ×200. (B) TUNEL apoptosis assay of blastocysts (green). Each sample was counterstained with DAPI to visualize DNA (blue). The original magnification was ×200. (C) Number of apoptotic cells in each blastocyst. *p<0.05.

As described above, the knockdown of *Nlrp2* with target-specific siRNA in oocytes or zygotes led to early developmental arrest. However, the effect of overexpression in zygotes is unknown. To determine this, zygotes were microinjected with pIRES2-AcGFP1-Nuc-Nlrp2 (pIRES2-Nlrp2), a bicistronic vector independently expressing NLRP2 and green fluorescent protein (GFP) (see Materials and Methods). Zygotes microinjected with pIRES2-Nlrp2 or pIRES2-AcGFP1-Nuc (pIRES2) reached the blastocyst stage and expressed GFP (Fig. 11A). The rates of blastocyst formation were similar between the two groups (Fig. 11B). As shown in Figure 11C, the *Nlrp2* transcripts were reduced dramatically and were undetectable after the 2-cell stage in the control group. However, the expression of *Nlrp2* persisted during preimplantation development in the group microinjected with pIRES2-Nlrp2. As expected, the level of NLRP2 protein in the pIRES2-Nlrp2-microinjected group gradually increased during preimplantation stages compared with the control group (Fig. 11D). To confirm whether accumulation of NLRP2 protein would influence cell fate and differentiation in blastocysts, we investigated the expression levels of OCT3/4 and CDX2, markers of pluripotency and trophectoderm differentiation [32], [33], [34], [35], respectively. The final cell fate and differentiation was not affected with regard to the expression of these markers (Fig. 12A). We also examined whether accumulation of NLRP2 protein would lead to apoptosis in blastocysts. We found that the number of apoptotic blastomeres in the pIRES2-Nlrp2 microinjected group was significantly increased compared with the control group (Fig. 12B, C).

## Discussion

The *Nlrp* gene family plays a pivotal role in the innate immune [36], [37], [38], [39], [40], [41], [42], [43], [44] and reproductive systems [3], [22], [45], [46], [47], [48] in mammals. *Nlrp5* (*Mater*) and *Nlrp14* are members of this family and are required for early embryonic development [3], [22]. We report here that *Nlrp2*, another member of this family, is a newly identified maternal effect gene. It is essential for female fertility, because developmental competence is dramatically compromised in *Nlrp2* knockdown oocytes and zygotes. Our data demonstrate that maternal depletion of *Nlrp2* blocks early embryogenesis in the mouse.

Maternal effect genes encode proteins that are produced during oogenesis and play a role during early embryogenesis. We demonstrated here that the temporal and spatial patterns of *Nlrp2* expression were developmentally regulated in a manner typical of maternal effect genes. *Nlrp2* mRNA appeared to accumulate during oogenesis and degrade after zygotic genome activation. Hence, the source of embryonic Nlrp2 transcripts is maternal and does not come from expression of the zygotic genome during preimplantation development. NLRP2 proteins were detected at different stages of follicle development, suggesting that the *Nlrp2* gene is involved in transcription–translation coupling and might play a role during folliculogenesis. After the 2-cell stage in embryos, *Nlrp2* mRNA was undetectable, but the protein persisted through to the blastocyst stage. The expression patterns of *Nlrp2* mRNA and its protein in parthenogenetic embryos were identical to those in normally fertilized embryos, suggesting that *Nlrp2* might be involved in two different developmental processes, but fulfill the same function.

The NLRP2 protein was distributed predominantly in the cytoplasm both in normal fertilized embryos and parthenogenetic embryos as shown by confocal microscopy. Immunogold electron microscopy demonstrated that the protein was present in the cytoplasm and close to nuclear pore complexes. In addition, immunogold particles were detected within the nucleus. However, the NLRP2 sequence does not display a nuclear localization signal. It is likely that other proteins interacting with NLRP2 mediate its transport into the nucleus; indeed, this protein contains leucine-rich repeat domains, which are known to participate in protein–protein interactions [49], [50], [51]. The subcellular localization of NLRP2 protein differs from NLRP5 (MATER) in mouse [52] and bovine oocytes [53]. Such localization of NLRP2 in multiple organelles suggests its participation in different intracellular functions.

Mouse embryos with mutations in maternal effect genes are characterized by developmental arrest during oogenesis or embryogenesis. Among the known maternal effect genes, only *Formin-2^−/−^* and *basonuclin*-deficient oocytes cannot undergo normal maturation [8], [9]. Compared with *Formin-2* null and *basonuclin*-deficient oocytes, *Nlrp2* knockdown GV-stage oocytes could reach metaphase II and emit the first polar body, suggesting that *Nlrp2* is not required for the metaphase spindle positioning or migration. Moreover, *Nlrp2* deficiency in oocytes does not perturb RNA polymerase I- or -II-mediated transcription, as the disruption of these processes leads to abnormal oocyte maturation [8], [9], [54]. However, the parthenogenetic embryos derived from *Nlrp2* knockdown oocytes were mainly arrested at the 2-cell stage. Interestingly, the expression levels of *Nlrp4f*, *Nlrp5*, *Nlrp9c* and *Nlrp14* were not affected in *Nlrp2* knockdown oocytes, suggesting that the expression of other *Nlrp* genes was not able to compensate for the absence of *Nlrp2*. Furthermore, the predominant 2- and 8-cell stage arrest of embryos derived from *Nlrp2* knockdown zygotes suggests that this maternal factor functions in early embryonic development. Previous studies also indicated that targeted invalidation/depletion of *Nlrp5* and *Nlrp14* does not reveal any gene compensation. In addition, microinjection with the pIRES2-Nlrp2 vector into zygotes did not affect subsequent cell fate and differentiation in terms of the expression and localization of OCT3/4 or CDX2. However, overexpression of *Nlrp2* increased the incidence of apoptosis in blastocysts, implying that an excessive accumulation of NLRP2 protein might impair the viability of blastomeres or activate the pathway of cell apoptosis, because NLRP2 contains a pyridine domain at the amino terminal that is primarily involved in apoptosis [55], [56], [57].

There are several reproduction-related Nlrp genes with conserved functions in mammals and primates. *Nlrp5* (*NLRP5*) transcripts are detected in the ovary in the mouse [3], [20], bovine [53], [58], [59], pig [60], rhesus macaque monkeys [28], [48] and humans [61]. Moreover it has been demonstrated that *Nlrp5* plays key roles in early embryo development in the mouse [3] and rhesus macaque monkeys [28]. *Nlrp14* is continuously expressing in germ cells [62] and in vitro knockdown experiments in zygotes led to developmental arrest in the mouse [22]. Moreover mutations in the testis-specific *NLRP14* gene in men leads to spermatogenic failure [63], implying that these genes are conserved functionally in a wide range of mammalian reproductive systems. Germline mutations in *NLRP2* result in a familial imprinting disorder (Beckwith–Wiedemann Syndrome) in humans [64], suggesting that this gene might have a similar function in the establishment and/or maintenance of genomic imprinting/methylation in the mouse. The *Dnmt1o* mutation causes disrupted genomic imprinting in early mouse embryogenesis [17]. Moreover, a gene disruption study determined that *PGC7/Stella* is indispensable for the maintenance of methylation involved in epigenetic reprogramming after fertilization [11], [65], [66]. Thus, the function of *Nlrp2* might be similar to the maternal effect gene *Dnmt1o* and/or *PGC7/Stella* during early preimplantation development. However, the localization of *Nlrp2* in the preimplantation embryo is very different, so the molecular function of *Nlrp2* may be different from *Dnmt1o* and *PGC7/Stella*. Identification of proteins that interact with NLRP2 could provide insights into the roles of this maternal factor in regulating the critical stages of development from the oocyte to the preimplantation embryo.

In summary, our data demonstrate that *Nlrp2* is a maternal effect gene and required for early embryonic development in the mouse. The maternal depletion of *Nlrp2* in zygotes by RNAi results in early embryonic arrest. *Nlrp2* knockdown oocytes can progress through the metaphase of meiosis I and emit the first polar body; however, the development of parthenogenetic embryos derived from *Nlrp2* knockdown oocytes mainly arrest at the 2-cell stage. In addition, overexpression of *Nlrp2* in zygotes appears to permit normal development, but increases the incidence of apoptosis in blastocysts.

## Materials and Methods

### Ethics Statement

The experimental procedure was approved by the Animal Care Commission of the College of Veterinary Medicine, Northwest A&F University. Adult male and female ICR strain mice were purchased from the Experimental Animal Center of The Fourth Military Medical University (Xi'an, China). They were maintained on a 14/10 h light/dark cycle with free access to food and water in the Laboratory Animal Facility of the College of Veterinary Medicine, Northwest A&F University.

### Chemicals

All chemicals and reagents were purchased from Sigma-Aldrich (St. Louis, USA) unless stated otherwise. Sterile plastic ware was purchased from Nunclon (Roskilde, Denmark).

### Collection and Culture of Oocytes and Embryos

Oocytes at the GV stage were obtained from the ovaries of 8–10-week-old females injected with 10 IU of pregnant mare serum gonadotrophin (PMSG) to stimulate the growth of the follicles. Forty-eight hours after PMSG administration the ovaries were placed in Hepes-buffered KSOM medium (H-KSOM) [67] containing 0.1 mM 3-isobutyl-1-methyl-xanthine to inhibit resumption of meiosis and oocytes were released from the largest follicles by puncturing them with hypodermic needle. The oocytes were completely freed of attached cumulus cells by repeated mouth pipetting.

Mature oocytes arrested in metaphase II were recovered from mice superovulated with intraperitoneal injections of PMSG and human chorionic gonadotrophin (hCG) 48 h apart. Metaphase II oocytes were collected from the oviduct ampullae at 16 h after the hCG injection. Cumulus masses were treated with hyaluronidase (1 mg/ml) to release ova. Metaphase II oocytes and cumulus cells were collected separately.

Zygotes (1-cell embryos) were obtained from females induced to superovulate as described above and mated with males immediately after hCG injection. Zygotes were collected from the oviducts 14 h after hCG injection. Cumulus cells surrounding zygotes were removed by brief exposure to hyaluronidase. Collection of embryos (2-cell, 4-cell, 8-cell, morula and blastocysts) was performed according to previously described protocols [30]. Embryos were cultured in groups of 30–50 in microdrops (100 µl) of KSOMaa medium [68] supplemented with 4 mg/ml bovine serum albumin (KSOMaa-BSA) under liquid paraffin oil in a humidified atmosphere of 5% CO_2_/95% air at 37°C.

### Parthenogenetic Activation of Oocytes

Metaphase II oocytes were subjected to parthenogenetic activation by exposure to activation medium (Calcium-free KSOMaa, 10 mM strontium chloride and 5 µg/ml cytochalasin B) for 6 h. The oocytes were washed in H-KSOM and cultured in KSOMaa-BSA. Pronuclear formation was scored and only activated embryos were used for quantitative RT–PCR, immunoblotting and immunofluorescence.

### Collection of Spermatozoa

Epididymides were removed from mature 12-week-old ICR male mice and punctured with hypodermic needles. The tissues were compressed to release spermatozoa into 1.5 ml polypropylene centrifuge tubes; 500 µl of H-KSOM medium was added to each tube. Then the tube was centrifuged (3000 rpm, 3 min) to collect spermatozoa.

### Cell Culture

RAW264.7 murine macrophage-like cells were maintained in RPMI 1640 medium (Gibco, Grand Island, NY, USA) supplemented with 10% fetal bovine serum (FBS, Gibco). Mouse D3 embryonic stem (D3 ES) cells were maintained in Dulbecco's modified Eagle's medium (DMEM, Gibco) supplemented with 20% FBS, 2 mM glutamine, 1% nonessential amino acids (Invitrogen, Carlsbad, CA, USA) and 0.1 mM β-mercaptoethanol. 1000 U/ml murine leukemia inhibiting factor (mLIF) was added to the culture medium to maintain ES cell pluripotency. Mouse F9 teratocarcinoma stem cells (F9 ES) were grown in DMEM supplemented with 10% FBS. Mouse D3 ES and F9 ES were plated on gelatinized multiwell tissue culture plates each containing 2 ml of medium. EMT6 mouse mammary carcinoma cells were cultured in Waymouth's medium (Invitrogen) supplemented with 15% FBS. All cell media were supplemented with 50 U/ml penicillin (Invitrogen) and 50 µg/ml streptomycin (Invitrogen). Cells were cultured at 37°C in a humidified 5% CO_2_/95% air incubator.

### RNA Isolation and Quantitative Real-Time RT–PCR

Total RNA extracts from 4-week-old mouse tissues (ovary, uterus, testis, kidney, lung, heart, liver, brain, stomach, small intestine, muscle and spleen) and different cells (RAW264.7, D3 ES, F9 ES, EMT6, cumulus cells and spermatozoa) were purified with RNeasy Mini Kits (Qiagen, Valencia, CA, USA). cDNA synthesis was performed using PrimeScript II 1st Strand cDNA Synthesis Kit (TaKaRa, Otsu, Japan).

Pooled oocytes or embryos (10 per group) were lysed and first-strand cDNA directly was synthesized using SuperScript® III CellsDirect cDNA Synthesis Kit (Invitrogen) according to the manufacturer's protocol. Lysis and reverse transcription were performed in the same tube. DNase I was added to eliminate genomic DNA prior to first strand synthesis. After synthesis, the first-strand cDNAs were amplified with specific primers by quantitative (q)RT–PCR. The primer pairs used for qRT–PCR are shown in Table 1. Each primer pair was confirmed by performing electrophoresis and melting temperature analysis of the PCR product to ensure its correct size and the absence of primer dimers. For negative controls, deionized water replaced cDNA in the real-time reaction tubes. The mRNA levels were quantified using SYBR Premix ExTaq™ II (TaKaRa) on an ABI PRISM 7700 Sequence Detection System (Applied Biosystems, Inc., Carlsbad, CA, USA). Samples were denatured at 95°C, 1 min and then subjected to 40 cycles of amplification (95°C, 5 s; 60°C, 30 s). Each data point was the average of duplicate assays performed on three independently obtained samples and transcript levels were calculated relative to the transcription of the housekeeping gene β-actin in every sample. Fold changes for each gene were calculated using the 2^−ΔΔCT^ method [69].

**Table 1:** Primer sequences for quantitative real-time PCR.

Genes	Primer sequences (5′-3′)	*T*_ann_ a (°C)
Nlrp2	F^b^: AACACTGAGCCTGAAACACTTGGA	60
	R^c^: CAGTTCAGTGGAGTGATGGAGCA	
Nlrp4f	F: CTTGAACCAGGCAGAGTGCAAC	60
	R: TGCCAAATTAAGAACCTTCAACGAC	
Nlrp5	F: CCAGAGCAGGAGCAGACATC	60
	R: TTCCAATCCACGTGCTTTCA	
Nlrp9c	F: CGCAATTGTACACACATTATCCAAG	60
	R: TCAGGCATATAAGTGTTGGTCTCAG	
Nlrp14	F: TTCTCAGCGCAAGGACTTAACTTTC	60
	R: AGTCTTTGTCCTCACTCACGGTTTC	
β-actin	F: GAAGTGTGACGTTGACATCCG	60
	R: ACTTGCGGTGCACGATGGAGG	

a Annealing temperature.

b Forward primer.

c Reverse primer.

### In Situ Hybridization

Ovaries from 3-week-old mice were fixed in 4% paraformaldehyde in 0.1 M PBS for 1 h before dehydration and paraffin wax embedding and sectioning (6 µm). Hybridization was carried out with Nlrp2 ISH Detection Kits (Boster-Bio, MK-3516-m, Wuhan, China) according to the manufacturer's instructions. The digoxigenin (DIG)-labeled oligonucleotide sequences used for in situ hybridization were 5′–GTGACTCTCTTTATCCAAGCACTGGGGCAGTGGTC–3′ 5′–GGCCAGAAGTTCTGCTTCCATTTAGCTGTGTATCT–3′ 5′–GGAGGGCCATGCAGCACAATTGCATGAGCAAATGG–3′ for *Nlrp2*. After deparaffinization and rehydration, sections were permeabilized with proteinase K (Boster-Bio) for 30 s and washed three times in 0.1 M PBS. Then the sections were postfixed in 1% paraformaldehyde for 10 min, prehybridized for 4 h at 42°C and hybridized overnight at 42°C. After hybridization, the sections were rinsed in 2× SSC buffer for 10 min at 37°C, 0.5× SSC for 15 min and twice in 0.2× SSC for 20 min at 37°C. Hybridized probes on sections were detected immunologically: sections were labeled with biotinylated anti-DIG antibody (Boster-Bio) and detected with Avidin, biotinylated peroxidase and DAB substrate solution (Boster-Bio) for 20–30 min at room temperature (RT). Control sections had the *Nlrp2* probes omitted. Hybridization signals were examined using a Nikon Eclipse Ti-S microscope equipped with a 198 Nikon DS-Ri1 digital camera (Nikon, Tokyo, Japan).

### Immunohistochemistry

Ovaries were collected from 3-week-old mice and fixed in 4% paraformaldehyde overnight, dehydrated through graded alcohol solutions and embedded in paraffin wax. Six-micrometer sections were deparaffinized in xylene, rehydrated through graded alcohol solutions and heated in 10 mM sodium citrate buffer (pH 6.0) with microwaves (15 min at medium power) and cooled to room temperature for antigen retrieval. Sections were then incubated with 10% nonimmune goat serum (Maixin-Bio, Kit-7770, Fuzhou, China) to block nonspecific sites followed by incubation with the NLRP2 polyclonal antibody (Sigma-Aldrich, SAB3500325) in blocking solution (1∶200 dilution of rabbit anti-NLRP2 antibody; Beyotime, P0102, Jiangsu, China) overnight at 4°C. For enzymatic staining, sections were incubated for 10 min at RT with a biotinylated goat anti-rabbit secondary antibody (Maixin-Bio). Following three 5 min washes in PBS buffer with 0.1% Triton X-100 (PBST), sections were incubated at RT for 10 min with streptavidin-linked alkaline phosphatase (Maixin-Bio) and then washed three times for 5 min each in PBST. 5-bromo-4-chloro-3-indolyl phosphate/nitro blue tetrazolium (BICP/NBT) substrate solution (Maixin-Bio) was applied to the sections for 10–30 min and the reaction was stopped by rinsing in PBS. Sections were then mounted with AEC Mounting Solution (Maixin-Bio, AEC-0038). Negative controls consisted of identical reactions with the omission of the primary antibody.

### Immunoblotting

To determine protein levels, fifty oocytes or embryos were loaded per lane. Total protein contents were separated by SDS-polyacrylamide gel (8%) and transferred to a PVDF membrane (Millipore, Bedford, MA) using semi-dry transfer. The membrane was blocked for 4 h at RT in Tris-buffered saline (TBS pH 7.4)/0.1% Tween 20 containing 5% dry milk following by an incubation overnight at 4°C with an anti-NLRP2 antibody (Sigma-Aldrich) at a 1∶1000 dilution, After several washes in TBS/Tween 20, membranes were incubated for 2 h at RT with a horseradish peroxidase-linked secondary antibody goat anti-rabbit (Pierce, Rockford, IL, USA) at a 1∶2000 dilution. The membrane was washed five times in TBS/Tween 20. Finally, the enhanced chemiluminescence (ECL) Advance Western Blotting detection system (Pierce) was used according to the manufacturer's instructions. Detection of actin, with anti-β actin polyclonal rabbit antibody (1∶1000, Santa Cruz Biotechnology Inc., sc-130656, Santa Cruz, CA, USA) and secondary anti-rabbit antibody (1∶5000, Pierce) was used as a loading control.

### Immunofluorescence

Oocytes and embryos were fixed in 4% paraformaldehyde in PBS for 45 min and permeabilized in PBS, 0.2% Triton X-100 for 10 min at RT. After a treatment with 3% BSA in PBS for 1 h, samples were incubated with the primary antibody diluted in PBS containing 1% BSA overnight at 4°C. Rabbit anti-NLRP2 antibodies (Sigma) were diluted 1∶100, anti-CDX2 rabbit polyclonal IgG (Santa Cruz, sc-134468) and anti-OCT3/4 mouse monoclonal antibody (Santa Cruz, sc-5279) was diluted 1∶50, respectively. After washing in PBS containing 0.3% polyvinylpyrrolidone (PBS/0.3% PVP), samples were incubated 1 h at RT with the secondary antibodies of Cy3-labeled goat anti-rabbit-IgG (Beyotime, A0516) for NLRP2, Alexa Fluor 555-labeled goat anti-rabbit IgG (Beyotime, A0452) for CDX2 and Alexa Fluor 488-labeled goat anti-mouse IgG (Beyotime, A0428) for OCT3/4. The secondary antibodies were diluted 1∶500 with PBS containing 1% BSA. After washing in PBS/0.3% PVP, nuclei were stained with DAPI (Beyotime, C1005) for 5 min. Negative controls were determined by processing samples as described above in the absence of primary antibody. Samples were observed using a Nikon eclipse Ti-S microscope (Nikon) or a Zeiss LSM510 confocal microscope equipped with differential interference contrast optics (Carl Zeiss, Inc., Thornwood, NY).

### Immunogold electron microscopic analysis

Ovaries from 10-day-old mice were fixed in 2.5% glutaraldehyde in 0.1 M phosphate buffer (PB) overnight at 4°C and washed thrice in fresh 0.1 M PB and then transferred into 1% osmium tetroxide in 0.1 M PB for 2 h at 4°C. After dehydrating in graded acetone, the ovaries were embedded in epoxy resin (Durcupan ACM, FLUKA, Buchs, Switzerland) and sectioned (70 nm) with a diamond knife mounted in an Ultracut microtome (Leica, Mannheim, Germany). The sections were mounted on nickel grids for immunogold reactions as described [52]. Briefly, the sections were subjected to blocking buffer and then incubated overnight at 4°C in droplets of rabbit anti-NLRP2 antibody (1∶500, Sigma-Aldrich). At the end of incubation, the sections were washed thrice to remove excess primary antibody and incubated again in blocking buffer and then incubated with droplets of the gold-labeled (10 nm) goat anti-rabbit IgG (1∶50; Sigma-Aldrich, G7402) for 2 h at RT. After several washings in 0.1 M PB and deionized water, the sections were counterstained with 2% uranyl acetate and lead citrate. Negative controls consisted of samples not incubated with the primary antibody. The sections were observed with a JEM-1230 electron microscope (JEOL Ltd., Tokyo, Japan).

### GV-stage oocyte and zygote electroporation with custom siRNA

Electroporation was used to introduce siRNA into mouse oocytes and zygotes as described [30]. GV-stage oocytes or zygotes were collected and washed in H-KSOM, then incubated in prewarmed acid Tyrode's solution for 12 s to weaken the zona pellucida [29]. They were then washed twice in fresh H-KSOM and kept in the pre-made KSOMaa drops. Afterwards, the siPORT *Amine* Transfection Agent (Ambion, AM4502, Austin, TX, USA) was diluted in Opti-MEM® I (Invitrogen) medium and incubated for 10 min at RT. The negative control siRNA (Ambion, Silencer® negative control #1, AM4611) or the custom-made *Nlrp2* siRNA (mixture of three target-specific 19–25 nt siRNA designed to knock down mouse *Nlrp2* expression; Santa Cruz, sc-149811) was diluted into Opti-MEM® I (Invitrogen) medium. The diluted negative control siRNA or the diluted custom-made *Nlrp2* siRNA were combined with the diluted siPORT *Amine* Transfection Agent and incubated for 10 min at RT. The GV-stage oocytes or zygotes were mixed with the transfection complexes. Simultaneously, the cell fusion instrument (BTX Inc., ECM 2001; San Diego, CA) was switched on. The GV-stage oocytes or zygotes were linearly arranged in a flat electrode chamber (BTX Inc.) and electroporated with the negative control siRNA (100 nM) and the *Nlrp2* siRNA in different concentrations (20, 40 and 60 nM). The electroporation condition was DC 20 volts/1 ms pulse length/3 pulses/0 repeats. The blank siPORT™ electroporation buffer was used as electroporation control (EP control). The negative control siRNA was used as siRNA control (Control siRNA).

Following electroporation, the GV-stage oocytes were washed thrice and cultured in MEM media supplemented with 3 mg/ml BSA. The maturation rates of oocytes were calculated after 24 h in vitro culture. Some mature oocytes were processed for qRT–PCR and immunoblotting, others were subjected to parthenogenetic activation and the subsequent development was analyzed. After electroporation, zygotes were washed thrice and cultured in KSOMaa-BSA. Some embryos were collected at 2 h (early 1-cell), 11 h (later 1-cell), 20 h (early 2-cell), 29 h (mid 2-cell) and 38 h (later 2-cell) post-electroporation for qRT–PCR. Some were collected at 4 h (1-cell), 28 h (2-cell) and 52 h (8-cell) post-electroporation for immunoblotting.

### Construction of NLRP2 expression vector and microinjection

Nlrp2 full-length CDS (3141 bp from 75 to 3215) with Sal I and Xma I sites in the 5′ and 3′ ends was obtained through PCR amplified (sense primer 5′–GCAGTCGACCATGGAACATTTTGATCCCCT–3′ antisense primer 5′–ATCCCGGGTTAAAAAATGAAATGAGGAAG–3′). The PCR product was digested with Sal I and Xma I and inserted to the Sal I/Xma I-digested pIRES2-AcGFP1-Nuc (Clontech) eukaryotic expression vector. The recombinant expression plasmid was then sequenced.

Zygotes were placed into a micro-culture dish at RT and microinjected with pIRES2-AcGFP1-Nuc-Nlrp2 (pIRES2-Nlrp2). Microinjection was performed using a Narishige microinjector connected to a micromanipulation system (Nikon, Tokyo, Japan). Approximately 5 pl of 0.8 µg/µl solution of expression plasmid was injected into the cytoplasm of per zygote. Zygotes were kept in H-KSOM during injection and then cultured in KSOMaa-BSA. Zygotes injected with pIRES2-AcGFP1-Nuc (pIRES2) were used as control. Embryos were collected for qRT–PCR and immunoblotting assays at different stages.

### TUNEL labeling assay

TUNEL (TdT-mediated dUTP nick end labeling) apoptosis assays were carried out with the DeadEnd™ Fluorometric TUNEL System (Promega, Madison, WI) according to the manufacturer's instructions. Apoptosis assays of blastocysts were performed as described [70].

### Statistical analysis

The results are presented as the mean ± SEM. Data were analyzed by one-way ANOVA and LSD tests using the SPSS 13.0 software (SPSS Inc., Chicago, IL, USA). The difference was considered statistically significant at P<0.05.

## References

[1] Latham KE (1999). Mechanisms and control of embryonic genome activation in mammalian embryos.. Int Rev Cytol.

[2] Christians E, Davis AA, Thomas SD, Benjamin IJ (2000). Maternal effect of Hsf1 on reproductive success.. Nature.

[3] Tong ZB, Gold L, Pfeifer KE, Dorward H, Lee E (2000). Mater, a maternal effect gene required for early embryonic development in mice.. Nat Genet.

[4] Nusslein-Volhard C, Lohs-Schardin M, Sander K, Cremer C (1980). A dorso-ventral shift of embryonic primordia in a new maternal-effect mutant of Drosophila.. Nature.

[5] Schupbach T, Wieschaus E (1986). Germline autonomy of maternal-effect mutations altering the embryonic body pattern of Drosophila.. Dev Biol.

[6] Bowerman B (1998). Maternal control of pattern formation in early Caenorhabditis elegans embryos.. Curr Top Dev Biol.

[7] Moody SA, Bauer DV, Hainski AM, Huang S (1996). Determination of Xenopus cell lineage by maternal factors and cell interactions.. Curr Top Dev Biol.

[8] Ma J, Zeng F, Schultz RM, Tseng H (2006). Basonuclin: a novel mammalian maternal-effect gene.. Development.

[9] Leader B, Lim H, Carabatsos MJ, Harrington A, Ecsedy J (2002). Formin-2, polyploidy, hypofertility and positioning of the meiotic spindle in mouse oocytes.. Nat Cell Biol.

[10] Gurtu VE, Verma S, Grossmann AH, Liskay RM, Skarnes WC (2002). Maternal effect for DNA mismatch repair in the mouse.. Genetics.

[11] Payer B, Saitou M, Barton SC, Thresher R, Dixon JP (2003). Stella is a maternal effect gene required for normal early development in mice.. Current Biology.

[12] Wu X, Viveiros MM, Eppig JJ, Bai Y, Fitzpatrick SL (2003). Zygote arrest 1 (Zar1) is a novel maternal-effect gene critical for the oocyte-to-embryo transition.. Nat Genet.

[13] Burns KH, Viveiros MM, Ren Y, Wang P, DeMayo FJ (2003). Roles of NPM2 in chromatin and nucleolar organization in oocytes and embryos.. Science.

[14] Bultman SJ, Gebuhr TC, Pan H, Svoboda P, Schultz RM (2006). Maternal BRG1 regulates zygotic genome activation in the mouse.. Genes Dev.

[15] Li L, Baibakov B, Dean J (2008). A subcortical maternal complex essential for preimplantation mouse embryogenesis.. Dev Cell.

[16] Zheng P, Dean J (2009). Role of Filia, a maternal effect gene, in maintaining euploidy during cleavage-stage mouse embryogenesis.. Proc Natl Acad Sci U S A.

[17] Howell CY, Bestor TH, Ding F, Latham KE, Mertineit C (2001). Genomic imprinting disrupted by a maternal effect mutation in the Dnmt1 gene.. Cell.

[18] Ting JPY, Conti BJ, Davis BK, Zhang JH, O'Connor W (2005). CATERPILLER 16.2 (CLR16.2), a novel NBD/LRR family member that negatively regulates T cell function.. Journal of Biological Chemistry.

[19] Harton JA, Linhoff MW, Zhang J, Ting JP (2002). Cutting edge: CATERPILLER: a large family of mammalian genes containing CARD, pyrin, nucleotide-binding and leucine-rich repeat domains.. J Immunol.

[20] Tong ZB, Nelson LM, Dean J (2000). Mater encodes a maternal protein in mice with a leucine-rich repeat domain homologous to porcine ribonuclease inhibitor.. Mamm Genome.

[21] Ohsugi M, Zheng P, Baibakov B, Li L, Dean J (2008). Maternally derived FILIA-MATER complex localizes asymmetrically in cleavage-stage mouse embryos.. Development.

[22] Hamatani T, Falco G, Carter MG, Akutsu H, Stagg CA (2004). Age-associated alteration of gene expression patterns in mouse oocytes.. Hum Mol Genet.

[23] Dade S, Callebaut I, Paillisson A, Bontoux M, Dalbies-Tran R (2004). In silico identification and structural features of six new genes similar to MATER specifically expressed in the oocyte.. Biochem Biophys Res Commun.

[24] Evsikov AV, Graber JH, Brockman JM, Hampl A, Holbrook AE (2006). Cracking the egg: molecular dynamics and evolutionary aspects of the transition from the fully grown oocyte to embryo.. Genes Dev.

[25] Svoboda P, Stein P, Hayashi H, Schultz RM (2000). Selective reduction of dormant maternal mRNAs in mouse oocytes by RNA interference.. Development.

[26] Wianny F, Zernicka-Goetz M (2000). Specific interference with gene function by double-stranded RNA in early mouse development.. Nat Cell Biol.

[27] Kim MH, Yuan X, Okumura S, Ishikawa F (2002). Successful inactivation of endogenous Oct-3/4 and c-mos genes in mouse preimplantation embryos and oocytes using short interfering RNAs.. Biochem Biophys Res Commun.

[28] Wu X (2009). Maternal depletion of NLRP5 blocks early embryogenesis in rhesus macaque monkeys (Macaca mulatta).. Hum Reprod.

[29] Grabarek JB, Plusa B, Glover DM, Zernicka-Goetz M (2002). Efficient delivery of dsRNA into zona-enclosed mouse oocytes and preimplantation embryos by electroporation.. Genesis.

[30] Wang H, Ding T, Brown N, Yamamoto Y, Prince LS (2008). Zonula occludens-1 (ZO-1) is involved in morula to blastocyst transformation in the mouse.. Dev Biol.

[31] Bruey JM, Bruey-Sedano N, Newman R, Chandler S, Stehlik C (2004). PAN1/NALP2/PYPAF2, an inducible inflammatory mediator that regulates NF-kappaB and caspase-1 activation in macrophages.. J Biol Chem.

[32] Beck F, Erler T, Russell A, James R (1995). Expression of Cdx-2 in the mouse embryo and placenta: possible role in patterning of the extra-embryonic membranes.. Dev Dyn.

[33] Palmieri SL, Peter W, Hess H, Scholer HR (1994). Oct-4 Transcription Factor Is Differentially Expressed in the Mouse Embryo during Establishment of the First 2 Extraembryonic Cell Lineages Involved in Implantation.. Developmental Biology.

[34] Mitsui K, Tokuzawa Y, Itoh H, Segawa K, Murakami M (2003). The homeoprotein Nanog is required for maintenance of pluripotency in mouse epiblast and ES cells.. Cell.

[35] Strumpf D, Mao CA, Yamanaka Y, Ralston A, Chawengsaksophak K (2005). Cdx2 is required for correct cell fate specification and differentiation of trophectoderm in the mouse blastocyst.. Development.

[36] Kanneganti TD, Ozoren N, Body-Malapel M, Amer A, Park JH (2006). Bacterial RNA and small antiviral compounds activate caspase-1 through cryopyrin/Nalp3.. Nature.

[37] Boyden ED, Dietrich WF (2006). Nalp1b controls mouse macrophage susceptibility to anthrax lethal toxin.. Nat Genet.

[38] Eisenbarth SC, Colegio OR, O'Connor W, Sutterwala FS, Flavell RA (2008). Crucial role for the Nalp3 inflammasome in the immunostimulatory properties of aluminium adjuvants.. Nature.

[39] Gross O, Poeck H, Bscheider M, Dostert C, Hannesschlager N (2009). Syk kinase signalling couples to the Nlrp3 inflammasome for anti-fungal host defence.. Nature.

[40] Duewell P, Kono H, Rayner KJ, Sirois CM, Vladimer G (2010). NLRP3 inflammasomes are required for atherogenesis and activated by cholesterol crystals.. Nature.

[41] Guarda G, Dostert C, Staehli F, Cabalzar K, Castillo R (2009). T cells dampen innate immune responses through inhibition of NLRP1 and NLRP3 inflammasomes.. Nature.

[42] Nakahira K, Haspel JA, Rathinam VA, Lee SJ, Dolinay T (2011). Autophagy proteins regulate innate immune responses by inhibiting the release of mitochondrial DNA mediated by the NALP3 inflammasome.. Nat Immunol.

[43] Vandanmagsar B, Youm YH, Ravussin A, Galgani JE, Stadler K (2011). The NLRP3 inflammasome instigates obesity-induced inflammation and insulin resistance.. Nat Med.

[44] Fontalba A, Gutierrez O, Fernandez-Luna JL (2007). NLRP2, an inhibitor of the NF-kappaB pathway, is transcriptionally activated by NF-kappaB and exhibits a nonfunctional allelic variant.. J Immunol.

[45] Murdoch S, Djuric U, Mazhar B, Seoud M, Khan R (2006). Mutations in NALP7 cause recurrent hydatidiform moles and reproductive wastage in humans.. Nat Genet.

[46] Zhang P, Dixon M, Zucchelli M, Hambiliki F, Levkov L (2008). Expression analysis of the NLRP gene family suggests a role in human preimplantation development.. PLoS One.

[47] Tian X, Pascal G, Monget P (2009). Evolution and functional divergence of NLRP genes in mammalian reproductive systems.. BMC Evol Biol.

[48] McDaniel P, Wu X (2009). Identification of oocyte-selective NLRP genes in rhesus macaque monkeys (Macaca mulatta).. Mol Reprod Dev.

[49] Kobe B, Deisenhofer J (1995). A structural basis of the interactions between leucine-rich repeats and protein ligands.. Nature.

[50] Kajava AV (1998). Structural diversity of leucine-rich repeat proteins.. J Mol Biol.

[51] Kobe B, Kajava AV (2001). The leucine-rich repeat as a protein recognition motif.. Curr Opin Struct Biol.

[52] Tong ZB, Gold L, De Pol A, Vanevski K, Dorward H (2004). Developmental expression and subcellular localization of mouse MATER, an oocyte-specific protein essential for early development.. Endocrinology.

[53] Pennetier S, Perreau C, Uzbekova S, Thelie A, Delaleu B (2006). MATER protein expression and intracellular localization throughout folliculogenesis and preimplantation embryo development in the bovine.. BMC Dev Biol.

[54] Dumont J, Million K, Sunderland K, Rassinier P, Lim H (2007). Formin-2 is required for spindle migration and for the late steps of cytokinesis in mouse oocytes.. Dev Biol.

[55] Bertin J, DiStefano PS (2000). The PYRIN domain: a novel motif found in apoptosis and inflammation proteins.. Cell Death Differ.

[56] Martinon F, Hofmann K, Tschopp J (2001). The pyrin domain: a possible member of the death domain-fold family implicated in apoptosis and inflammation.. Current Biology.

[57] Kohl A, Grutter MG (2004). Fire and death: the pyrin domain joins the death-domain superfamily.. C R Biol.

[58] Pennetier S, Uzbekova S, Perreau C, Papillier P, Mermillod P (2004). Spatio-temporal expression of the germ cell marker genes MATER, ZAR1, GDF9, BMP15,andVASA in adult bovine tissues, oocytes, and preimplantation embryos.. Biol Reprod.

[59] Ponsuksili S, Brunner RM, Goldammer T, Kuhn C, Walz C (2006). Bovine NALP5, NALP8, and NALP9 genes: assignment to a QTL region and the expression in adult tissues, oocytes, and preimplantation embryos.. Biol Reprod.

[60] Pisani LF, Ramelli P, Lazzari B, Braglia S, Ceciliani F (2010). Characterization of maternal antigen that embryos require (MATER/NLRP5) gene and protein in pig somatic tissues and germ cells.. J Reprod Dev.

[61] Tong ZB, Bondy CA, Zhou J, Nelson LM (2002). A human homologue of mouse Mater, a maternal effect gene essential for early embryonic development.. Hum Reprod.

[62] Horikawa M, Kirkman NJ, Mayo KE, Mulders SM, Zhou J (2005). The mouse germ-cell-specific leucine-rich repeat protein NALP14: a member of the NACHT nucleoside triphosphatase family.. Biol Reprod.

[63] Westerveld GH, Korver CM, van Pelt AM, Leschot NJ, van der Veen F (2006). Mutations in the testis-specific NALP14 gene in men suffering from spermatogenic failure.. Hum Reprod.

[64] Meyer E, Lim D, Pasha S, Tee LJ, Rahman F (2009). Germline mutation in NLRP2 (NALP2) in a familial imprinting disorder (Beckwith-Wiedemann Syndrome).. PLoS Genet.

[65] Bortvin A, Goodheart M, Liao M, Page DC (2004). Dppa3/Pgc7/stella is a maternal factor and is not required for germ cell specification in mice.. BMC Dev Biol.

[66] Nakamura T, Arai Y, Umehara H, Masuhara M, Kimura T (2007). PGC7/Stella protects against DNA demethylation in early embryogenesis.. Nat Cell Biol.

[67] Biggers JD, McGinnis LK, Raffin M (2000). Amino acids and preimplantation development of the mouse in protein-free potassium simplex optimized medium.. Biol Reprod.

[68] Lawitts JA, Biggers JD (1991). Optimization of mouse embryo culture media using simplex methods.. J Reprod Fertil.

[69] Livak KJ, Schmittgen TD (2001). Analysis of relative gene expression data using real-time quantitative PCR and the 2(−Delta Delta C(T)) Method.. Methods.

[70] Su JM, Wang YS, Li YY, Li RZ, Li Q (2011). Oxamflatin Significantly Improves Nuclear Reprogramming, Blastocyst Quality, and In Vitro Development of Bovine SCNT Embryos.. PLoS One.

